# BMEFIQA: Blind Quality Assessment of Multi-Exposure Fused Images Based on Several Characteristics

**DOI:** 10.3390/e24020285

**Published:** 2022-02-16

**Authors:** Jianping Shi, Hong Li, Caiming Zhong, Zhouyan He, Yeling Ma

**Affiliations:** College of Science and Technology, Ningbo University, Ningbo 315300, China; shijianpingky@126.com (J.S.); zhongcaiming@nbu.edu.cn (C.Z.); mayeling@nbu.edu.cn (Y.M.)

**Keywords:** blind quality assessment, multi-exposure fused images, structure, naturalness, colorfulness

## Abstract

A multi-exposure fused (MEF) image is generated by multiple images with different exposure levels, but the transformation process will inevitably introduce various distortions. Therefore, it is worth discussing how to evaluate the visual quality of MEF images. This paper proposes a new blind quality assessment method for MEF images by considering their characteristics, and it is dubbed as BMEFIQA. More specifically, multiple features that represent different image attributes are extracted to perceive the various distortions of MEF images. Among them, structural, naturalness, and colorfulness features are utilized to describe the phenomena of structure destruction, unnatural presentation, and color distortion, respectively. All the captured features constitute a final feature vector for quality regression via random forest. Experimental results on a publicly available database show the superiority of the proposed BMEFIQA method to several blind quality assessment methods.

## 1. Introduction

In view of the increasing development of image processing technologies, it has become more feasible to help humans perceive the real world in high-quality images. For example, multi-exposure fusion (MEF) and high dynamic range (HDR) imaging both belong to image enhancement technology, which can provide excellent detail information and an ideal, natural appearance [1]. HDR imaging technology requires additional processing, such as generation, image conversion to a low dynamic range image, and visualization on common displays. Unfortunately, all of these procedures can produce artifacts that affect the quality of HDR images [2]. Multi-exposure fusion technology is relatively simple, it does not need intermediate operations, and it has also been applied in some practical fields [3]. However, the unique process of integration from multiple different exposure images into one ultimate image will result in distortion due to the fusion weighting assignment. Obviously, the distortion will influence human perception and result in different human opinion scores. Hence, to measure the distortion degree objectively, it is necessary to develop a quality assessment method for MEF images.

In recent years, many researchers have developed some image quality assessment (IQA) methods [4,5,6,7,8,9,10,11,12,13,14]. Those methods can be categorized into full-reference (FR) methods, reduced-reference (RR) methods, and no-reference (NR) methods. The FR and RR methods both need original image information to make a comparison; however, it is opposite to the truth that there is never a pre-defined reference in the real application. Therefore, it is more urgent to design NR methods for IQA tasks.

Based on the considerations of the inherent characteristics of MEF images, a new NR-IQA method is proposed in this paper to predict the quality of MEF images more accurately, and it is named BMEFIQA.

The main contributions are detailed as follows:

(1) Inspired by the various characteristics of MEF images, structural, naturalness, and colorfulness features are extracted from different standpoints to perceive their distortion.
(2) On account of structure loss produced by abnormal exposure, the exposure map is weighted to the gradient similarity to detect structural distortion.
(3) The experimental results demonstrate that the proposed method is competent for MEF images and superior to several NR-IQA methods.

The remainder of this paper is constructed as follows: In Section 2, related works are presented. In Section 3, the proposed BMEFIQA method is described in detail. The experimental results and analysis based on a public MEF image database are presented in Section 4. Finally, the conclusion is drawn in Section 5.

## 2. Related Works

Currently, there are many efficient NR-IQA methods for ordinary images. For instance, Moorthy et al. [5] proposed an NR-IQA method named the distortion identification-based image verity and integrity evaluation (DIIVINE), which is based on natural scene statistics (NSSs). In [6], Saad et al. utilized an NSS model of discrete cosine transform coefficients to design the IQA method, which is referred to as BLINDS-II. In [7], Mittal et al. extracted features from the empirical distribution of locally normalized luminance and the products of locally normalized luminance in the spatial domain; the method is named BRISQUE. Liu et al. [8] constructed the CurveletQA method by utilizing curvelet transform to extract a set of statistical features. Xue et al. [9] combined the gradient magnitude map with the Laplacian of Gaussian response to perceive the structural information of images and showed highly competitive performance, dubbed as GradLog. Fang et al. [10] employed the degree of deviation from NSS models to represent the unnatural character of contrast-distorted images, which is termed ContrastQA. Li et al. [11] proposed an NR-IQA method based on structural degradation, which is described by the gradient-weighted histogram of local binary pattern (LBP) calculation on the gradient map (GWH-GLBP). Liu et al. [12] developed the oriented gradient (OG) IQA method by studying the quality relevance of the relative gradient orientation. Gu et al. [13] combined local and global considerations to design an NR-IQA method called NIQMC. Oszust [14] captured the information carried by image derivatives of different orders by local features and used it for image quality prediction. Zhang et al. [15] integrated the features of natural image statistics and created a multivariate Gaussian model of image patches for quality assessment. Xu et al. [16] proposed an NR-IQA method based on high-order statistics aggregation, which needs a small codebook.

Except for the above IQA methods for ordinary images, some studies have also contributed to predicting the quality of tone-mapped images. For example, Gu et al. [17] devised an effective blind tone-mapped quality index (BTMQI) via the analysis of information, naturalness, and structure. Kundu et al. [18] derived the HDR image gradient-based evaluator (HIGRADE) based on standard measurements of the bandpass and newly conceived differential NSS. Although the above IQA methods for ordinary and tone-mapped images showed good performance, they are not appropriate for the prediction of the quality of MEF images, as their distortion type is distinguished from the above two types of images.

For MEF images, there also exist some IQA methods, which belong to FR methods. For instance, Zheng et al. [19] proposed a quantitative metric called the ratio of spatial frequency error; this is derived from the definition of spatial frequency, which reflects local intensity variation. Ma et al. [20] proposed an objective method based on the principle of structural similarity and a novel measure of patch structural consistency. Xing et al. [21] combined contrast information with structural similarity and saturation similarity to predict the quality of an MEF image. Fang et al. [22] utilized a pyramid subband contrast preservation scheme and an information theory-adaptive pooling strategy to establish a quality assessment method for MEF images of both static and dynamic scenes. Deng et al. [23] designed a method by extracting color, texture, and structural features. Martinez et al. [24] utilized the multi-scale scheme to compute structural similarities. Considering the limitation of such FR methods, which need reference information, an NR method was proposed in our previous work [25]. However, there is room for improvement. Therefore, to predict the quality of an MEF image more accurately, its characteristics should be taken into consideration more comprehensively.

## 3. The Proposed BMEFIQA Method

**Figure 1:**
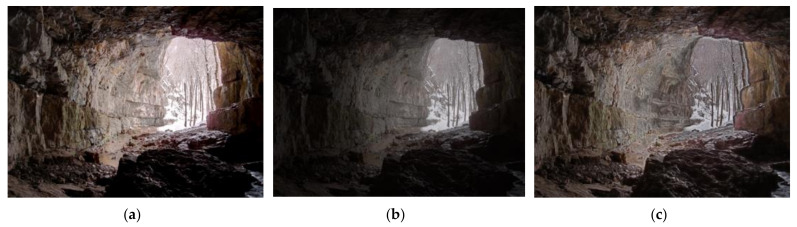
An example of multi-exposure fusion images generated by three MEF algorithms. (**a**) MEF image generated by Merten’s algorithm [27] (MOS = 7.6957); (**b**) MEF image generated by Raman’s algorithm [28] (MOS = 4.2609); (**c**) MEF image generated by local energy weighting [29] (MOS = 4.3043).

Since MEF images are fused by multiple images with different exposure levels [26], the weight assignment process will introduce some artifacts, such as detail loss, structural degradation, unnaturalness, and color distortion. Figure 1 gives a vivid presentation of MEF images generated by three different MEF algorithms, which show the different visual effects. Figure 1a, generated by Merten’s algorithm [27], has the highest mean opinion score (MOS) and is the best quality. The detailed information is well preserved, except for the over-exposed part (i.e., outside the entrance of the cave). Moreover, it also has much more abundant color information than the others, and it seems more natural. At a first glance of Figure 1b, which is generated by Raman’s algorithm [28], the blackened scene is the initial perception of humans. In fact, the brightness decline also leads to detail and color information loss. The MEF image generated by local energy weighting [29] shows proper brightness, but it loses the original naturalness, and it also introduces some artifacts around the wall and stones. Therefore, structural, naturalness, and color information are the main factors that influence the visual quality of MEF images.

**Figure 2:**
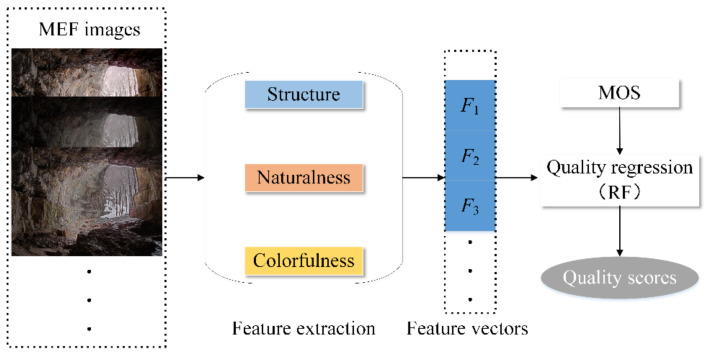
The framework of the proposed BMEFIQA method.

To fill the gap of quality prediction for MEF images under the condition with a lack of reference information, a novel method named BMEFIQA is proposed in this paper, which considers three factors (i.e., structure, naturalness, and colorfulness). Figure 2 presents the pipeline of the proposed BMEFIQA method. Specifically, three types of quality-sensitive features corresponding to the above three factors are extracted to generate overall feature vectors. Then, random forest is utilized to train a quality regression model, thus aggregating all excavated features. More details are given in the following subsections.

### 3.1. Structural Features

Structural information usually carries the basic visual content of a scene, and the human visual system (HVS) has strong adaptability to extract the structure for visual perception [30]. For an MEF image, distortion introduction will always destroy its structural information, such as abnormal exposure (i.e., over-exposure or under-exposure). Therefore, the visual quality of an MEF image can be determined by measuring whether the structural information is damaged or not, especially for over-exposed and under-exposed regions.

First, the exposure of the MEF image needs to be measured to distinguish the regions of over-exposure and under-exposure. Given MEF image ***I*** and converting it into a gray-scale one, its corresponding exposure map ***E***_I_ can be calculated by measuring the distance between its normalized pixel intensity and the constant 0.5. Specifically, when the normalized pixel intensity is close to 0 or 1, the corresponding pixel is regarded as under-exposed or over-exposed. Therefore, ***E***_I_ can be defined as

(1) $$E_{I}=1-\exp\frac{{\left(I_{y}-0.5\right)}^{2}}{2\tau^{2}}$$

where ***I***_y_ is the normalized pixel intensity of the gray-scaled MEF image ***I***, and *τ* is the standard variance of the Gaussian function, which is set to 0.2 according to previous experience [31].

**Figure 3:**
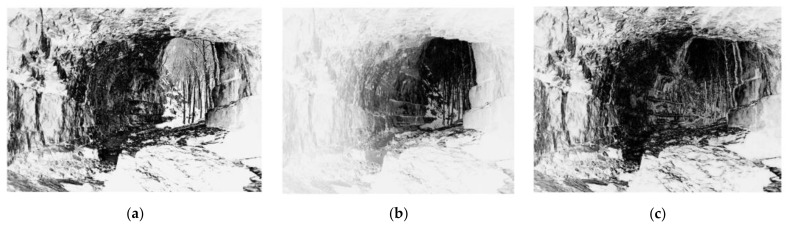
(**a**–**c**) The corresponding exposure maps of the MEF images in Figure 1.

Figure 3 shows three exposure maps of the MEF images in Figure 1. By comparing with Figure 1, it can be observed that the brighter regions are the under-exposed or over-exposed regions, and the fainter regions represent the normal exposed regions in Figure 3.

As an effective structural information feature, the gradient is utilized to describe the structure loss phenomenon. To further measure the influence of abnormal exposure on the acquisition of gradient information, some fake MEF images are obtained by darkening and brightening the real MEF images, which are denoted as ***I***_f_. They can be produced as follows:

(2) $$I_{f}=I_{r}\cdot C$$

where *C* = *c*, *c*∈{1/3.5, 1/5, 1/6.5, 3.5, 5, 6.5}, and ***I***_r_ represents the gray-scaled MEF image ***I***. After that, six fake MEF images can be generated.

Then, gradient maps ***G*** of such fake MEF images are calculated as

(3) $$G=\sqrt{{\left(I_{f}⊗p_{x}\right)}^{2}+{\left(I_{f}⊗p_{y}\right)}^{2}}$$

where *p_x_* and *p_y_* are the Prewitt filters, along with the horizontal and vertical directions, respectively. ⊗ indicates the convolution operator. Additionally, the gradient map of the real MEF image ***I*** is also obtained by (3), which is denoted as ***G***_I_.

Then, the gradient similarities between ***I*** and the generated six fake MEF images are calculated. Let ***G***_s_ be the similarities of ***G*** and ***G***_I_, which are calculated to quantify the influence of abnormal exposure. They are defined as

(4) $$G_{s}=\frac{2G\cdot G_{I}+C_{1}}{G^{2}+G_{I}{}^{2}+C_{1}}$$

where *C*_1_ is a constant to avoid zero denominators, which is set to 10^−8^.

Since the distortions of the over-exposed and the under-exposed regions are easier for the human eye to perceive, combining the gradient similarities ***G***_s_ with the exposure map ***E***_I_ can make the distortion perception more accurate. Therefore, gradient combined with exposure weighting is defined as

(5) $$G_{e}=\frac{\sum_{k}\sum_{f}G_{s}\cdot E_{I}}{\sum_{k}\sum_{f}E_{I}}$$

where *k* and *f* are the indexes of the horizontal and vertical pixels.

As the same number of fake MEF images, after combining six gradient similarity maps with ***E***_I_, the obtained *G*_e_ is a 6-dimensional feature that describes the structure loss of the MEF image.

From the statistical perspective, the NSS model is utilized in the gradient domain to represent the structural variation in the MEF images. Specifically, the gradient map ***G***_I_ is processed by the local mean subtraction and divisive normalization to obtain the mean subtracted contrast normalized (MSCN) coefficients [7], which are expressed by

(6) $${\hat{G}}_{I}(i,j)=\frac{G_{I}(i,j)-\mu (i,j)}{\delta (i,j)+1}$$

where ${\hat{G}}_{I}(i,j)$ are the MSCN coefficients of ***G***_I_ at the position of (*i*, *j*). *μ*(*i*, *j*) and *δ*(*i*, *j*) are the local mean and standard deviation of ***G***_I_(*i*, *j*), respectively.

Different degrees of structural distortion will inevitably affect the distribution of the MSCN coefficients of ***G***_I_. As the distribution has a Gaussian-like appearance, a generalized Gaussian distribution (GGD) is utilized to match the MSCN coefficients, and the mathematical expression is given by

(7) $$f(l,\alpha ,\sigma^{2})=\frac{\alpha }{2\beta \Gamma \left(1/\alpha \right)}\cdot e^{-{\left(\left|l\right|/\beta \right)}^{\alpha }}$$

where $\beta =\sigma \sqrt{\Gamma \left(1/\alpha \right)\Gamma \left(3/\alpha \right)}$, $\Gamma \left(\cdot \right)$ represents the gamma function. The parameters *α* and *σ*^2^ represent the shape and variance of the Gaussian distribution, respectively. Therefore, the two parameters *α* and *σ*^2^ are taken as the 2-dimensional global structural features.

In addition, the pairwise products of neighboring MSCN coefficients are also calculated to capture the relationship of the neighboring pixels. The MSCN coefficients are processed in four directions, and this process is expressed as

(8) $$\begin{array}{l} H\left(i,j\right)={\hat{G}}_{I}(i,j){\hat{G}}_{I}(i,j+1)\\ V\left(i,j\right)={\hat{G}}_{I}(i,j){\hat{G}}_{I}(i+1,j)\\ D_{1}\left(i,j\right)={\hat{G}}_{I}(i,j){\hat{G}}_{I}(i+1,j+1)\\ D_{2}\left(i,j\right)={\hat{G}}_{I}(i,j){\hat{G}}_{I}(i+1,j-1) \end{array}$$

where *H*(*i*, *j*), *V*(*i*, *j*), *D*_1_(*i*, *j*), and *D*_2_(*i*, *j*) are the results processed along with the horizontal, vertical, main diagonal, and sub-diagonal directions, respectively.

Then, an asymmetric generalized Gaussian distribution (AGGD) is utilized to fit each pairwise product, which is defined as

(9) $$f\left(k;v,\omega_{l}^{2},\omega_{r}^{2}\right)=\left\{\begin{array}{l} \frac{v}{\left(\beta_{l}+\beta_{r})\Gamma (v^{-1}\right)}\exp\left(-{\left(\frac{-k}{\beta_{l}}\right)}^{v}\right),k<0\\ \frac{v}{\left(\beta_{l}+\beta_{r})\Gamma (v^{-1}\right)}\exp\left(-{\left(\frac{-k}{\beta_{r}}\right)}^{v}\right),k\geq 0 \end{array}\right.$$

(10) $$\varphi =\left(\beta_{r}-\beta_{l}\right)\frac{\Gamma \left(2/v\right)}{\Gamma \left(1/v\right)}$$

where $\beta_{l}=\omega_{l}\sqrt{\Gamma \left(v^{-1}\right)/\Gamma \left(v^{-3}\right)}$ and $\beta_{r}=\omega_{r}\sqrt{\Gamma \left(v^{-1}\right)/\Gamma \left(v^{-3}\right)}$.

The above parameters *v*, *φ*, $\omega_{l}^{2}$, and $\omega_{r}^{2}$ constitute the compensation features that perceive the global structural distortion. As a result, the compensation features calculated in the four directions form a 16-dimensional feature vector.

However, the phenomena of structure loss also contain the loss of detailed information, which can be measured via entropy. Block entropy is calculated to perceive the local detail information variation, which is denoted as *e*_b_. After calculating the local entropy in 8 × 8 blocks of each MEF image, the entropy calculation is used to measure the distribution of the obtained local entropy, which can be expressed as

(11) $$e=-\sum_{n=1}^{m}p(e_{b}(n))\log_{2}p(e_{b}(n))$$

where *m* is the block number of each MEF image, and *p*(·) is the probability density of the *m*-th block entropy value.

Furthermore, the mean value and standard deviation of the block entropy are also calculated to measure the overall detail loss, which are denoted as *e*_m_ and *e*_s_, respectively. Finally, *e*, *e*_m_, and *e*_s_ are integrated to form another set of 3-dimensional structural features.

### 3.2. Naturalness Features

Generally, a high-quality MEF image has a natural-like appearance. MEF algorithms may disrupt the natural statistical regularities in the spatial domain. From a global perspective, the naturalness can be quantified via the NSS-based model. An MEF image, ***I***, is converted to a gray-scale image, ***I***_r_, and then the MSCN coefficients of ***I***_r_ are calculated according to Equation (6). Moreover, the GGD model defined in Equation (7) is utilized to capture the statistical property, and the shape and variance parameters are taken as the first group of global naturalness features.

From another perspective, naturalness is also affected by the overall brightness and contrast, which is seriously influenced by under-exposed and over-exposed conditions. It has been demonstrated that the mean and standard deviation values of the image intensity can represent the brightness and contrast of an image [32]; the entropy can also describe the distortion of brightness. Therefore, these moment features (mean and standard deviation) and the entropy feature are applied to build NSS models to capture the naturalness variation undergoing the distortions. Among them, Gaussian probability density functions are used to fit the mean and standard deviation. The specific definitions are as follows:

(12) $$d_{a}=\frac{1}{\sqrt{2\pi }\xi_{a}}\exp\left[-\frac{{\left(a-\tau_{a}\right)}^{2}}{2\xi_{a}^{2}}\right]$$

(13) $$d_{s}=\frac{1}{\sqrt{2\pi }\xi_{s}}\exp\left[-\frac{{\left(s-\tau_{s}\right)}^{2}}{2\xi_{s}^{2}}\right]$$

where *a* and *s* are the mean and standard deviation values, respectively. *d_a_* and *d_s_* are the possibilities of the MEF image being natural when *a* and *s* are given. The parameters in Equations (12) and (13) are set to $\xi_{a}$ = 26.063, $\tau_{a}$ = 118.559, $\xi_{s}$ = 12.858, and $\tau_{s}$ = 57.274, respectively.

In addition, the entropy feature is fitted by the extreme value probability density function, which is defined as

(14) $$d_{o}=\frac{1}{\xi_{o}}\exp\left[\frac{o-\tau_{o}}{\xi_{o}}-\exp\left[\frac{o-\tau_{o}}{\xi_{o}}\right]\right]$$

where *o* is the entropy value of ***I***_r_. *d_o_* is the possibility of the MEF image being natural when *o* is given. $\xi_{o}$ and $\tau_{o}$ are the parameters that are set to 0.258 and 7.54, respectively.

As a result, *d_a_*, *d_s_*, and *d_o_* form the second group of global naturalness features. Combined with the first group of features, 5-dimensional features are used to describe the naturalness of the MEF image.

### 3.3. Colorfulness Features

Abundant color information is important for an outstanding MEF image, as it illustrates that the image has proper color saturation and realistic scene chroma. The weight assignment processes in the different MEF algorithms may emphasize different parts of the image content, as different humans do not always focus on the same things. As Figure 1 shows, different emphasis brings different distortion. Nevertheless, the color distortion measure is necessary and right. In previous work, it was proved that the color perception of human vision is mainly processed in the opponent color space [33]. Therefore, the opponent color space is obtained by the red–green–blue (RGB) color channels in the RGB color space. The transformation processes are expressed as *T*_1_ = *R* − *G*, *T*_2_ = (*R* + *G*)/2 − *B*; *T*_1_ is the obtained red–green channel, and *T*_2_ is the obtained yellow–blue channel.

A combination of the two transformed channels is utilized to represent the colorfulness of the MEF image. The specific definition is as follows:

(15) $$C_{t}=\log\left(\gamma_{T_{1}}^{2}/{\left|a_{T_{1}}\right|}^{0.2}\right)\cdot \log\left(\gamma_{T_{2}}^{2}/{\left|a_{T_{2}}\right|}^{0.2}\right)$$

where $\gamma_{T_{1}}^{2}$ and $\gamma_{T_{2}}^{2}$ denote the variances in *T*_1_ and *T*_2_, respectively. $a_{T_{1}}$ and $a_{T_{2}}$ denote the mean value of *T*_1_ and *T*_2_, respectively.

In Figure 1, it can be seen that over-exposure and under-exposure will both affect the representation of excellent color with a decrease in contrast. Therefore, the contrast energies are calculated in the opponent color space to perceive the color distortion. Let *C_e_* be the calculated contrast energy; it is obtained by

(16) $$C_{e}=\frac{\varepsilon \cdot \rho \left(I_{g}\right)}{\rho \left(I_{g}\right)+\varepsilon \cdot \chi }-\vartheta_{g}$$

where $g\in \{T_{1},T_{2}\}$; $I_{T}{}_{{}_{1}}$ and $I_{T}{}_{{}_{2}}$ denote the red–green and yellow–blue channels of ***I***, respectively. $\rho \left(I_{g}\right)=\sqrt{{\left(I_{g}⊗p_{x}\right)}^{2}+{\left(I_{g}⊗p_{y}\right)}^{2}}$; χ is the contrast gain to correct and normalize all filter responses, ε is the maximum value of $\rho \left(I_{g}\right)$, and $\vartheta_{g}$ is the noise threshold. χ and $\vartheta_{g}$ are fixed at the same settings as in [34].

Finally, the mean value of *C_e_* is calculated as another feature except for *C_t_*. In fact, according to the opponent color space, two channels can be used to perform the contrast energy calculation process, and 2-dimensional features are obtained.

Moreover, to obtain a more complete sense of color distortion, the NSS model is also executed in the two opponent color channels. Specifically, *T*_1_ and *T*_2_ are processed with the MSCN coefficient calculation defined in Equation (6) and the GGD model defined in Equation (7), respectively. The obtained shape and variance parameters constitute 4-dimensional complementary color features.

### 3.4. Feature Aggregation and Quality Regression

So far, 39-dimensional features are extracted via structure, naturalness, and colorfulness analyses, denoted as ***S***_F_, ***N***_F_, and ***C***_F_, respectively. According to the obtained quality-sensitive features, the mapping relationship between the features and human subjective scores should be learned to achieve the purpose of quality prediction. As an effective regression manner, random forest (RF) is used to pool the high-dimensional features to indicate the quality of the MEF image. The specific expression can be represented as follows:

(17) $$Q=\eta \left(F\right)$$

where *Q* is the final quality score of the MEF image; $\eta \left(\cdot \right)$ denotes the mapping function by RF; and *F* denotes the final all-feature vectors, *F* = {***S***_F_, ***N***_F_, ***C***_F_}.

## 4. Experiment Results

### 4.1. Experimental Protocol

**Figure 4:**
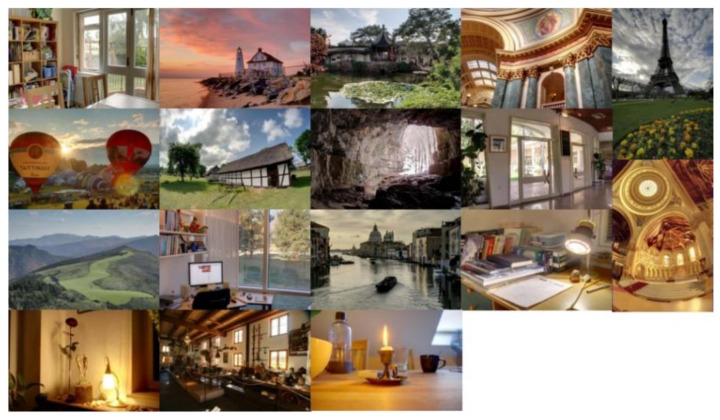
Source images in the benchmark MEF database [35].

In the experiment, an open MEF subjective assessment database [35] is selected to evaluate the performance of the proposed BMEFIQA method. The database consists of 136 MEF images, which portray different scenarios. The corresponding perceptual quality score is subjectively tested by numerous observers, that is, human subjective scores, and the MOS values of each MEF image are obtained from them. These MEF images are generated by distinct types of MEF algorithms, including local energy weighted linear combination, global energy weighted linear combination, Li12 [36], Raman09 [28], ShutaoLi12 [37], Mertens07 [27], Gu12 [29], and ShutaoLi13 [38]. Specifically, 136 MEF images are derived from 17 source multi-exposure images. The detailed contents are reported in Table 1 and Figure 4.

Three standard performance evaluation criteria are utilized to evaluate the performance of the proposed method, and they include the Person linear correlation coefficient (PLCC), Spearman’s rank-order correlation coefficient (SROCC), and root-mean-square error (RMSE). Specifically, PLCC, SROCC, and RMSE are used to evaluate the prediction accuracy, prediction monotonicity, and prediction error, respectively. Moreover, before the calculation of the PLCC value, a five-parameter logistic regression is used to perform nonlinear mapping between the predicted score and the subjective quality scores; the definition is given by

(18) $$Q_{f}=\lambda_{1}\left[\frac{1}{2}-\frac{1}{\exp\left(\lambda_{2}\left(Q-\lambda_{3}\right)\right)}\right]+\lambda_{4}Q+\lambda_{5}$$

where *Q_f_* is the fitted score, and *λ*_1_, *λ*_2_, *λ*_3_, *λ*_4_, and *λ*_5_ are the fitting parameters, which can be obtained via the *nlinfit* function in MATLAB software. The initial values of these five fitting parameters are guided by the video quality expert group (VQEG) [39]. The final values are determined by nonlinear least-squares optimization between the subjective quality scores and *Q_f_*. When calculating the PLCC value, the input (i.e., predicted score *Q*) should be replaced by *Q_f_*.

An excellent quality assessment method should always have higher PLCC and SROCC values and a lower RMSE value. To obtain a robust criterion, we employ 17-fold cross-validation to test the proposed BMEFIQA method. Specifically, the MEF image database is divided into 17 subsets, 16 of which are used for RF model training, and the remaining are used for testing. Each subset contains MEF images belonging to the same scene. Finally, we report the average criteria values (i.e., PLCC, SROCC, and RMSE) across all fold trails when the train/test cycles are over for all the scenes.

### 4.2. Performance Comparison

To verify the superiority of the proposed method, two types of the NR-based method, which has demonstrated effectiveness for other images, are used to make the performance comparison. The first type contains ten methods, which are designed for ordinary images, dubbed as 2D NRIQA, namely, DIIVINE [5], BLIND-II [6], BRISQUE [7], CurveletQA [8], GradLog [9], ConstrastQA [10], GLBP [11], OG [12], NIQMC [13], and SCORER [14]. The second type contains three methods designed for tone-mapped images, dubbed as TM-NRIQA, namely, BTMQI [17], HIGRADE-1 [18], and HIGRADE-2 [18]. All the comparison methods are learning-based ones following the 17-fold cross-validation to obtain their performance for MEF images, which is consistent with the proposed method. To ensure that the results are not biased, the performances of the comparison methods are all obtained by running the original released codes from the authors.

The overall performance comparison results are presented in Table 2. To highlight the best performance, the PLCC, SROCC, and RMSE values are shown in bold. Obviously, the proposed BMEFIQA method reveals its better performance compared to the other comparison methods, which verifies its validity. We can draw some observations from Table 2. On the one hand, most 2D NRIQA methods exhibit poor performance, except for GradLog; the worst performance indicators for PLCC and SROCC are only 0.163 and 0.113, respectively. The reasons for this phenomenon can be roughly summarized as follows: First, the distortion of an MEF image presents differently from an ordinary 2D image, such as difficult semantic understanding and color deterioration when suffering from over-exposure and under-exposure. Second, the GradLog method combines gradient amplitude maps with the Laplace of Gaussian responses to sense structural information, which can describe the structural distortion of MEF images well. Hence, its performance outperforms other comparison 2D NRIQA methods. On the other hand, compared with 2D NRIQA methods, TM-NRIQA methods achieve better performance. This can be attributed to the fact that the distortion of tone-mapped images seems similar to that of MEF images. However, such results should also be improved to predict the quality of MEF images more accurately. The proposed BMEFIQA method considers the structure, naturalness, and colorfulness of an MEF image from three aspects. Its comprehensive consideration advocates that it obtains superior performance to the competing methods.

**Figure 5:**
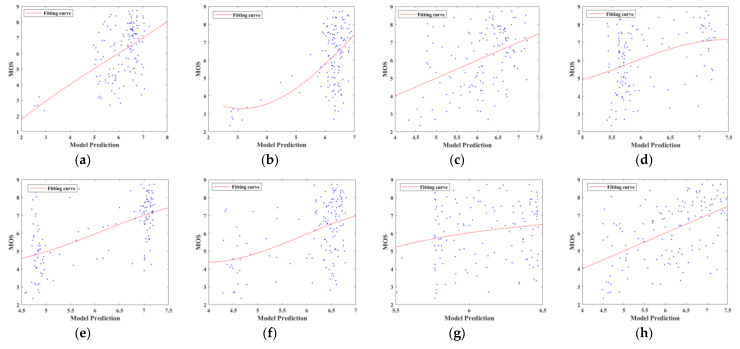

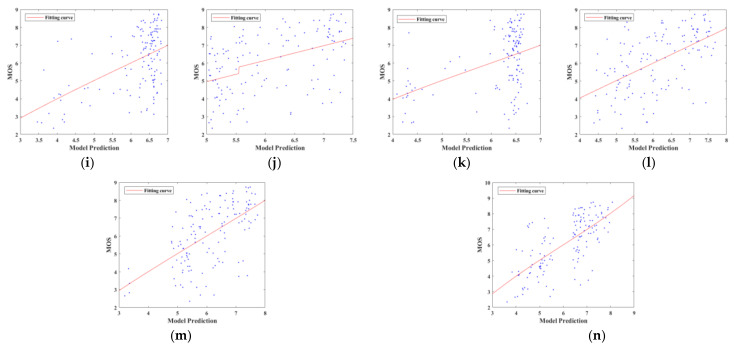
Scatter plots for the objective predicted scores and MOS values of sixteen NR-IQA methods. (**a**) DIIVINE [5]; (**b**) BLINDS_II [6]; (**c**) BRISQUE [7]; (**d**) CurveletQA [8]; (**e**) GradLog [9]; (**f**) ContrastQA [10]; (**g**) GWH-GLBP [11]; (**h**) OG [12]; (**i**) NIQMC [13]; (**j**) SCORER [14]; (**k**) BTMQI [17]; (**l**) HIGRADE-1 [18]; (**m**) HIGRADE-2 [18]; (**n**) Proposed.

In order to compare the performance of the different methods more intuitively, Figure 5 shows the scatter plots for the objective predicted scores and MOS values of fourteen NR-IQA methods. They are conducted using the whole MEF database, and the scatter points are fitted by the logistic function. The closer these scatter points are to the fitting line, the better the performance. In Figure 5, it can be seen that the predicted scores of the proposed method show a higher correlation with the MOS values than the other NR-IQA methods.

### 4.3. Impacts of Different Features and Block Sizes

Based on the above performance comparison, the proposed BMEFIQA method is demonstrated to have a good-quality prediction capability. However, for the individual features, their role in the overall method remains ambiguous. To realize their purpose, the individual features ***S***_F_, ***N***_F_, and ***C***_F_ and their different combinations are used to train the corresponding regression models to predict the quality of the MEF images separately. As shown in Table 3, the obtained regression models are written as Model-*t*, *t* ∈ {1, 2, …, 7}, which indicates the number of individual features and their possible combinations. Their respective performance results are also given in Table 3, and the best performance results are shown in bold. The following can be observed: First, among the individual features, compared with the naturalness features ***N***_F_ and color features ***C***_F_, the structural features ***S***_F_ show a relatively strong performance. For the overall performance, ***S***_F_ contributes the most. Second, among the different combinations, the combination of ***S***_F_ and ***C***_F_ produces the best performance. Although the performance of ***C***_F_ itself is relatively weak, when it is combined with ***N***_F_, its performance is improved, indicating that it provides a good auxiliary effect. Third, when all ***S***_F_, ***N***_F_, and ***C***_F_ features are incorporated together, the best performance can be obtained. Therefore, we believe that ***S***_F_, ***N***_F_, and ***C***_F_ features are complementary to each other.

In the block entropy calculation process, as different block sizes may affect the quality prediction performance, the block size should be determined to obtain the optimal performance. In the experiment, performances are tested under different block sizes, as shown in Table 4. Specifically, the block size varies from 8 to 64, and the corresponding PLCC, SROCC, and RMSE values are given. Additionally, the run time under different block sizes is also developed. From the results, we can observe that the PLCC and SROCC values and the run time decrease with an increase in block size. Under comprehensive consideration, the block size is set to 8.

### 4.4. Run Time

In a practical application, in addition to performance, efficiency is also important for the methods. Therefore, the mean implementation times for all MEF images in the MEF database of the proposed method and the other competing methods are reported in Table 5. All the original codes are implemented on a Windows 10 1.80 GHz Intel Core i7-8565U CPU with 8GB RAM and NVIDIA MX 150, using MATLAB 2017b. In Table 5, it can be seen that the proposed BMEFIQA method has a moderate execution time.

### 4.5. Discussion

In this study, a novel NR-IQA method that can handle the quality prediction task for MEF images is proposed. Specifically, the proposed BMEFIQA method considers the structure, naturalness, and colorfulness of MEF images, and it extracts the corresponding features of these three aspects. Through the performance comparison in the previous section, the proposed method shows its potential in predicting the quality of MEF images. It can be used in the application terminal to monitor quality with no reference information provided.

Although the proposed BMEFIQA method considers some image attributes, it is limited for kaleidoscopic images in real cases. They may be influenced by some special situations, which cannot be foreseen. As a result, regarding the performance of the proposed method, there is still room for improvement. For instance, the human attention mechanism will affect the perception of MEF image quality, and humans will have different artistic biases toward MEF images in different scenes. Deep learning is well known for its ability to automatically learn the features of some images. In future work, deep learning-based methods or more handcrafted features with the attention mechanism can be incorporated to improve the performance of the BMEFIQA method. Moreover, the MEF image database should be expanded for data training and testing to increase the robustness and practicability of the method.

## 5. Conclusions

This paper proposes a blind quality assessment method for multi-exposure fusion (MEF) images based on structure, naturalness, and colorfulness analyses of MEF images, named BMEFIQA. For the structure analysis, exposure map calculation is implemented to weight the gradient similarities, statistical modeling is used in the gradient domain, and entropy calculation is performed to characterize the loss of structural information. For the naturalness analysis, statistical modeling in the spatial domain combines the statistics of moment features and entropy, which are built to characterize scene naturalness. For colorfulness analysis, opponent color space is used to calculate contrast energy and build the statistical model. Finally, random forest is used to fuse the extracted features into prediction quality. Experiments on the public MEF image database demonstrate the superiority of the proposed BMEFIQA method.

## Figures and Tables

**Table 1:** The details of the MEF image database [35].

No.	Source Sequences	Size	Image Source
1	Balloons	339 × 512 × 9	Erik Reinhard
2	Belgium house	512 × 384 × 9	Dani Lischinski
3	Lamp1	512 × 384 × 15	Martin Cadik
4	Candle	512 × 364 × 10	HDR Projects
5	Cave	512 × 384 × 4	Bartlomiej Okonek
6	Chinese garden	512 × 340 × 3	Bartlomiej Okonek
7	Farmhouse	512 × 341 × 3	HDR Projects
8	House	512 × 340 × 4	Tom Mertens
9	Kluki	512 × 341 × 3	Bartlomiej Okonek
10	Lamp2	512 × 342 × 6	HDR Projects
11	Landscape	512 × 341 × 3	HDRsoft
12	Lighthouse	512 × 340 × 3	HDRsoft
13	Madison capitol	512 × 384 × 30	Chaman Singh Verma
14	Memorial	341 × 512 × 16	Paul Debevec
15	Office	512 × 340 × 6	Matlab
16	Tower	341 × 512 × 3	Jacques Joffre
17	Venice	512 × 341 × 3	HDRsoft

**Table 2:** The overall performance comparison results.

Metrics	PLCC	SROCC	RMSE
DIIVINE	0.491	0.403	1.452
BLINDS-II	0.534	0.346	1.409
BRISQUE	0.414	0.380	1.517
CurveletQA	0.371	0.337	1.548
GradLog	0.631	0.567	1.293
ContrastQA	0.458	0.412	1.482
GWH-GLBP	0.163	0.113	1.645
OG	0.523	0.525	1.421
NIQMC	0.519	0.404	1.425
SCORER	0.481	0.494	1.461
BTMQI	0.452	0.343	1.487
HIGRADE-1	0.561	0.566	1.380
HIGRADE-2	0.585	0.583	1.352
Proposed	**0.694**	**0.673**	**1.200**

**Table 3:** Performance comparison of different feature combinations.

Models	*S*_F_	*N*_F_	*C*_F_	PLCC	SROCC	RMSE
Model-1	√	×	×	0.646	0.506	1.272
Model-2	×	√	×	0.557	0.457	1.385
Model-3	×	×	√	0.389	0.416	1.535
Model-4	√	√	×	0.651	0.520	1.263
Model-5	√	×	√	0.658	0.640	1.255
Model-6	×	√	√	0.604	0.599	1.328
Model-7	√	√	√	**0.694**	**0.673**	**1.200**

**Table 4:** Performance comparison with different block sizes and the corresponding run time.

Block Size	PLCC	SROCC	RMSE	Time (s)
8 × 8	0.694	0.673	1.200	1.3383
16 × 16	0.677	0.655	1.227	0.4744
32 × 32	0.683	0.658	1.218	0.2857
64 × 64	0.688	0.659	1.210	0.2347

**Table 5:** The results of execution time by different methods.

Methods	DIIVINE	BLINDS_II	BRISQUE	CurveletQA
Time (s)	6.6024	14.7361	0.0414	2.5141
**Methods**	**GradLog**	**ContrastQA**	**GWH-GLBP**	**OG**
Time (s)	0.0343	0.0259	0.0576	0.0314
**Methods**	**NIQMC**	**SCORER**	**BTMQI**	**HIGRADE-1**
Time (s)	1.9241	0.5878	0.0758	0.2602
**Methods**	**HIGRADE-2**	**Proposed**		
Time (s)	1.9040	**1.3383**		

## Data Availability

Not applicable.
